# Effects of long-term functional training combined with blood flow restriction training on athletic performance and body composition in basketball athletes

**DOI:** 10.3389/fphys.2026.1789909

**Published:** 2026-05-08

**Authors:** Di Wang, Moran Lyu, Zhiheng Li, Ming Li, Yuliu Tao, Aisong Zhu

**Affiliations:** 1 Key Laboratory of Blood-Stasis-Toxin Syndrome of Zhejiang Province, Zhejiang Chinese Medical University, Hangzhou, China; 2 Zhejiang Engineering Research Center for “Preventive Treatment” Smart Health of Traditional Chinese Medicine, Zhejiang Chinese Medical University, Hangzhou, China; 3 School of Basic Medical Sciences, Zhejiang Chinese Medical University, Hangzhou, China; 4 School of Physical Education and Sports School, Soochow University, Suzhou, China; 5 School of Physical Education and Sport Science, Fujian Normal University, Fuzhou, China

**Keywords:** athletes, athletic performance, blood flow restriction, body composition, functional training

## Abstract

**Background:**

This study examined the effects of a 24-week functional training (FT) and blood flow restriction combined with FT (BFR-FT) on athletic performance and body composition in athletes.

**Methods:**

Twenty-six male basketball players were randomly assigned to either the experimental group (EG) or the control group (CG). The CG performed FT, while the EG performed BFR-FT, both for 24 weeks. Assessments were conducted at seven time points, every 4 weeks, measuring 1RM half-squat, 1RM squat, vertical jump, 30m sprint, agility, 17-return shuttle run, body weight, and limb circumference and skinfold thickness.

**Results:**

(1) Significant time × group interactions were observed for 1RM half−squat and speed (*P* < 0.01). The EG showed superior speed (weeks 12, 20, *P* < 0.01) and 1RM half−squat (week 8, *P* = 0.02; weeks 12–24, *P* < 0.01). For 1RM squat and endurance, both interaction and time effects were significant (*P* < 0.05). For explosive power and agility, only time effects were significant (*P* < 0.01). (2) Significant interactions were found for body weight (*P* = 0.04), thigh circumference (*P* < 0.01), and calf skinfold thickness (*P* = 0.02), and the main effects of time were also significant (*P* < 0.01). For calf circumference and thigh skinfold thickness, only time effects were significant (*P* < 0.01).

## 1 Introduction

In modern competitive sports, the continuous evolution of training theory and practice has led to the incorporation of an increasing array of scientifically validated training methods into athletes’ regimens, all designed to optimize performance and enhance athletic capabilities. Among these methods, functional training (FT) and blood flow restriction training (BFR) have garnered considerable attention in recent years, emerging as key approaches in both sports science and health promotion (Bieniec and Grabara, 2025; Lyu et al., 2024; Niknam et al., 2025; Pazokian et al., 2022).

Both FT and BFR have been extensively demonstrated to enhance daily exercise routines and rehabilitation therapies. FT addresses functional weaknesses through multi-muscle, multi-dimensional, and multi-joint exercises, thereby strengthening neuromuscular coordination and improving overall athletic performance (Yin and Yuan, 2015). On the other hand, BFR employs specialized equipment to restrict blood flow velocity in the limbs, slowing venous return to the heart. This results in a unique physiological stress stimulus, inducing an internal environment of hypoxia and ischemia. Consequently, metabolic byproducts and muscle-growth-promoting hormones are secreted in increased quantities, stimulating the development of type II muscle fibers, significantly enhancing muscle cross-sectional area and improving qualities such as strength and speed (Moritani et al., 1992; Yasuda et al., 2009; Zhao et al., 2019). Compared to traditional resistance training, FT is particularly beneficial in enhancing neuromuscular coordination and control (Yin and Yuan, 2015). In contrast, resistance training combined with BFR may compromise physical function, diminishing the precision, stability, and neuromuscular coordination of antagonistic muscles (Wu et al., 2026).

Based on the inherent characteristics of FT and BFR, Da Silva-Grigoletto et al. (2020) proposed the incorporation of BFR during FT. FT typically includes exercises performed in unstable positions, aimed at enhancing neuromuscular control and compensating for deficiencies in motor function. To ensure safety and minimize disruption to technical movements, training often utilizes lower external loads (Yin and Yuan, 2015). The integration of BFR with FT effectively mitigates the limitations of FT, which is often constrained by the difficulty of applying substantial external loads and the relatively weaker load stimulation inherent to FT. Researchers have suggested that incorporating BFR into FT can reduce blood flow velocity, increasing internal physiological load and further enhancing the training effects (Da Silva-Grigoletto et al., 2020; Lyu et al., 2023; 2024).

Research has shown that BFR-FT not only enhances muscle strength and increases follistatin levels while reducing myostatin in older adults, but it also improves lower-body muscle strength, speed, and anaerobic capacity in male soccer players (Lyu et al., 2023; Pazokian et al., 2022). Additionally, Lyu et al. (2024) found that low-load FT combined with BFR equally improves non-dominant lower limb athletic performance, lower limb function, and muscle mass in elite female soccer players as compared to high-intensity FT. In fact, some improvements were more pronounced, suggesting that this approach could serve as a substitute for high-load FT.

Although existing research has consistently shown that FT and BFR-FT enhance strength, speed, and other aspects of athletic performance, much of the literature has primarily focused on verifying these positive effects. Few studies have systematically examined, from a temporal dynamics perspective, how different training methods influence athletic performance and body composition over time. For instance, when do these interventions significantly affect various athletic abilities? At which stage do these changes reach their peak? What specific differences exist between FT and BFR-FT in enhancing performance and body composition? These questions remain largely unanswered. To address these gaps, the present study recruited male basketball players to undergo 24 weeks of either FT or BFR-FT. The primary aim was to investigate the effects of these interventions on athletic performance and body composition at various stages of the training period. The findings will provide valuable insights into the time-course patterns of how FT and BFR-FT influence different athletic abilities, enabling coaches to tailor training plans based on competition schedules and individual athlete characteristics. Moreover, the results will offer important reference data for future research in the field.

The research hypothesis posits that, when compared to traditional FT alone, BFR-FT is expected to result in significantly greater improvements in athletic performance and body composition among basketball players. Moreover, it is anticipated that BFR-FT will yield more rapid gains, thereby accelerating improvements in both athletic performance and body composition metrics.

## 2 Methods

### 2.1 Study design

This study utilized a randomized controlled trial (RCT) design to assess the effects of long-term FT and BFR-FT on athletic performance and body composition in basketball players. A total of 26 high-level male basketball players were recruited and randomly assigned to either the experimental group (EG), which underwent BFR-FT, or the control group (CG), which followed FT alone. Experimental measurements included One-Repetition Maximum (1RM) half-squat and squat, one-step approach vertical jump, 30m sprint, three-second agility drill, 17-return shuttle run, body weight, lower limb circumference, and lower limb skinfold thickness. Performance metrics and body composition were assessed at seven time points: pre-intervention, and at 4, 8, 12, 16, 20, and 24 weeks. The subjects were instructed to refrain from engaging in any additional resistance training beyond the study’s intervention and their regular training routines.Due to the nature of the intervention, the coaches responsible for administering the training could not be blinded. However, to ensure methodological rigor, blinding was applied to both the outcome assessors and data analysts. Double-blinding was achieved as follows: (1) all assessors remained blinded to group allocation throughout the testing period and did not participate in any aspect of the training intervention; (2) data analysts were blinded to group allocation until the completion of data analysis.

### 2.2 Subjects

Sample size estimation was performed using G*Power 3.1 software (Düsseldorf, Germany), with the following parameters: F tests → ANOVA: repeated measures, within–between interaction; *A priori* computation with α = 0.05, power = 0.80, and an effect size of f = 0.25. The minimum required sample size was 18 sbjects. Considering an estimated dropout rate of 20%, a total of 26 male national-level basketball players were included in the study. The effect size (f = 0.25) was selected as a medium effect based on widely accepted methodological standards in sport science, following Cohen’s (1988) general criteria. Inclusion criteria required that all subjects be in good health with no significant injuries. Exclusion criteria included inability to continue participation due to injury, illness, scheduling conflicts, or absence from more than three training sessions. A simple randomization procedure was used to allocate subjects to either the EG (n = 13) or CG (n = 13). Random numbers were generated using the RAND function in Microsoft Excel, and the allocation was completed based on a 1:1 ratio. The results were sealed in opaque, numbered envelopes, which were opened by the training instructors after baseline assessments. No statistically significant differences were observed between the two groups in terms of height, body weight, or age (**Table 1**). All subjects were fully informed of the experimental procedures and emergency protocols, voluntarily agreed to participate, and provided written informed consent. During the experiment, four athletes dropped out due to scheduling conflicts and personal reasons. The study was approved by the Institutional Review Board of Soochow University (approval No. SUDA20250327H09) and was conducted in accordance with the Declaration of Helsinki.

**Table 1:** Basic information of subjects.

Group	n	Height (cm)	Weight (kg)	Age (years)	Training experience (years)
EG	13	181 ± 6	73 ± 7	22 ± 2	5.1 ± 1.5
CG	13	182 ± 6	74 ± 7	21 ± 2	5.0 ± 1.6

### 2.3 Procedures

#### 2.3.1 Athletic performance test

One Repetition Maximum Strength. This study employed the Gymaware linear sensor system (Kinetic Performance Technology Pty Ltd, Australia) to conduct a load-progression testing protocol on the athlete subjects. The protocol aimed to assess their maximum strength performance during half-squat and squat movements. Maximal strength was estimated using linear regression statistics (Load = m + b ± Z, where Load represents maximal strength, m is velocity, b is the intercept, and Z is the error term) (Jovanović and Flanagan, 2014). During testing, the load applied during the half-squat and squat movements was observed alongside the velocity displayed by the sensors. The relationship between velocity and load was then used to determine the subjects’ maximal strength. A total of 3–5 test sets were conducted, with load increments of 20–30 kg between sets. To ensure test validity, the squat-to-stand speed in the first set must remain above 1 m/s, while the final set toward the end of testing requires reducing the squat-to-stand speed to below 0.5 m/s.

Explosive Power Test. This test is conducted in a standard indoor basketball facility. Prior to testing, the one-step approach vertical jump measuring device was calibrated to ensure measurement accuracy. Subjects, wearing basketball shoes, are required to perform a one−step approach from within 1.5 m of the vertical jump measuring device, then jump and reach the highest possible point with one arm. The test is conducted twice, and the best performance is recorded as the valid result for each subject.

Speed Test. This test is conducted on a standard athletics track to evaluate the subject’s 30-meter sprint speed. During the test, an optical photoelectric timing system (Swift Duo, Australia) is used to precisely time the subject at both the starting and finishing lines. All subjects must adopt a standing start position. Upon readiness, subjects initiate the start independently without external commands. After the start, the photoelectric timing system automatically captures and records the time taken for subjects to pass the starting and finishing lines. The entire test procedure is repeated twice, with the best result from the two trials serving as the subject’s valid data.

**Figure 1:**
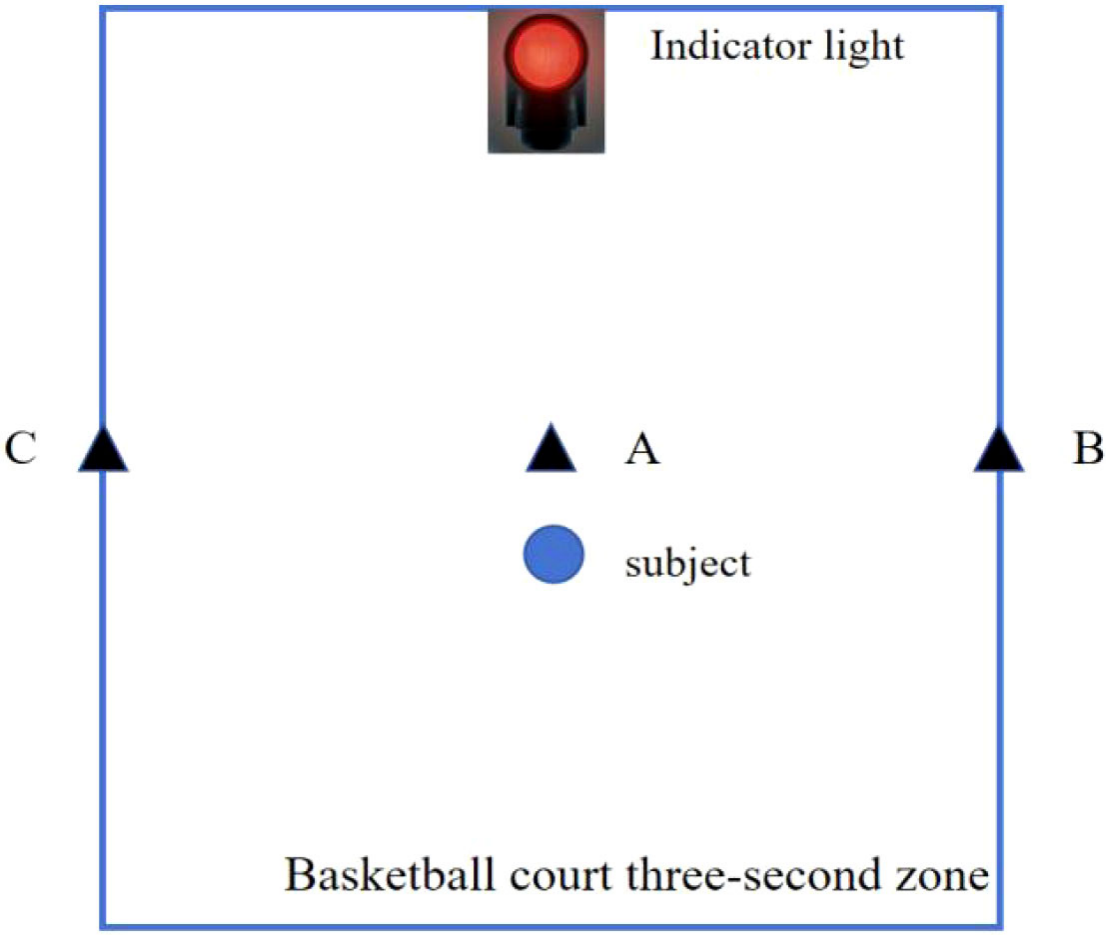
Schematic diagram of the 3-second shuttle agility test. Diagram of a basketball court three-second zone outlined in blue, showing a labeled indicator light at the top, three black triangles labeled **(A–C)** on the edges, and a blue circle labeled subject near the center.

Agility Test. The shuttle agility test is conducted within the three-second zone of a basketball court, as shown in **Figure 1**. The specific test procedure is designed as follows: The subject stands at the center of the three-second zone (Point A). Upon seeing the color of the indicator light held by the staff member in front (blue indicates left, red indicates right), the subject quickly runs to either Point C on the left or Point B on the right. After reaching the designated point, the subject immediately runs to the opposite point (Point B or Point C). Upon completing the run, the subject returns to Point A. The indicator light color changes randomly during the test. The best result from two attempts is recorded as the subject’s valid data.

Endurance Test. All participating athletes underwent a 17-return shuttle run test in the basketball gymnasium. Athletes were positioned in batches along the basketball court sideline. Upon the tester’s “Go” command, athletes sprinted from the starting point to the opposite sideline, immediately turned around, and repeated this shuttle run 17 times. Each set was followed by a 2h rest interval. A single test consisted of 4 sets, and the final score was the average of all 4. During the testing procedure, subjects were verbally encouraged by the examiner to exert maximal effort, thereby obtaining their most authentic endurance performance (Li et al., 2018).

#### 2.3.2 Body composition test

Weight Measurement. subjects must wear shorts and a short-sleeved shirt, be barefoot, have emptied their bowels and bladder, and refrain from eating, drinking, or engaging in strenuous physical activity. Weight measurements are taken in the morning (7:00-8:00) using an electronic scale (Shanghe SH-200, China). Athletes should stand relaxed and naturally in the center of the scale, maintaining a stable posture throughout the measurement.

Lower Limb Circumference Measurement. In the morning (7:00-8:00), the subjects were in a fasted state, with no prior physical activity and their lower limbs relaxed. The measurements of lower limb circumference were taken using an electronic caliper (manufactured in China by Shengtaixin, Model Dzjkc). For thigh circumference measurement, subjects stood naturally while the tape measure was kept horizontal, encircling the maximum circumference at the base of the thigh below the hip. For calf circumference, the measurement was taken at the midpoint between the patella and the ankle, identifying the point of maximum calf circumference. The tape measure was kept horizontal during measurement. The entire procedure was repeated twice, and the average of the two measurements was used as the valid data for the subject. If the discrepancy between the two measurements exceeded 5 mm, an additional measurement was taken to ensure that the difference was within 5 mm.

Skinfold Thickness Measurement. In the morning (7:00-8:00), subjects were in a fasted state, had not engaged in exercise, and were in a relaxed position. Skinfold thickness measurements were conducted using a caliper (Peacock LA-13, manufactured in Japan). For thigh skinfolds, measurements were taken at the midpoint between the patella and the inguinal crease, with the subject standing naturally. For calf skinfolds, the measurement was taken at the point of maximum circumference on the medial side of the calf, with the subject standing and the foot placed on a box, with the knee bent to approximately 90 degrees. The caliper was applied with the thumb and index finger pinching the skinfold, and the measurement was taken 1 cm below the fingers. The entire process was repeated twice, and the average of the two readings was recorded as the valid data. If the discrepancy between the two measurements exceeded 5 mm, an additional measurement was performed to ensure that the difference was within 5 mm. The measurement protocol followed the guidelines provided by the National Strength and Conditioning Association (NSCA) (Miller, 2012).

### 2.4 Intervention protocol

**Figure 2:**
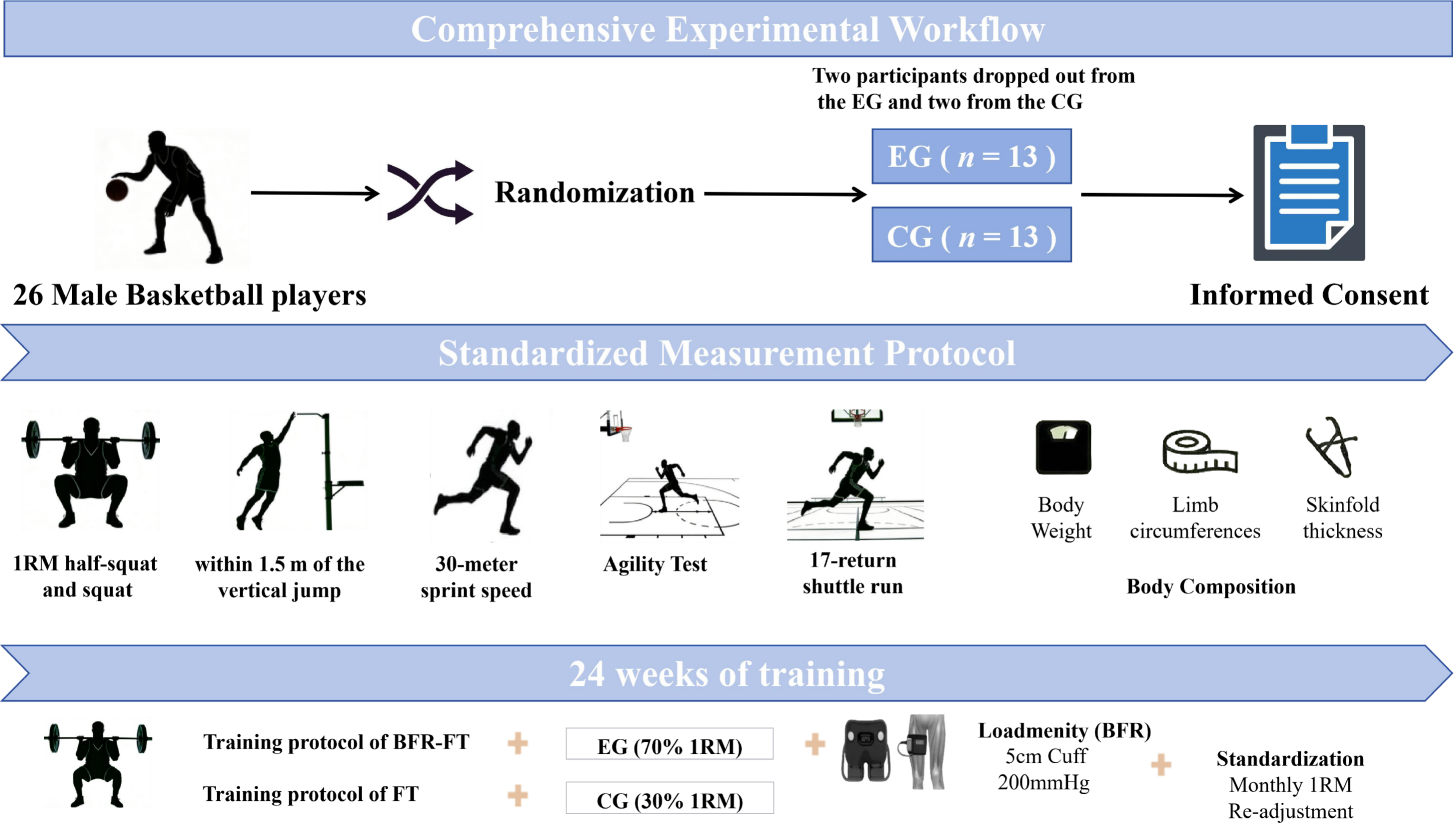
Comprehensive experimental workflow. Infographic depicting an experimental workflow with 26 male basketball players randomized into experimental and control groups, completing informed consent, undergoing body composition and physical tests, and participating in 24 weeks of specialized training protocols with standardization procedures.

The training protocol for this study was developed based on prior research and tailored to the specific characteristics of basketball (Lyu et al., 2023, 2024; Palmieri-Smith et al., 2022; Yin and Yuan, 2015). The complete experimental procedure is illustrated in **Figure 2**. The pressure value used for BFR during the intervention period was established with reference to previous studies (Cook et al., 2007; Che, 2021; Lyu et al., 2023; 2024).The training plan for the EG is detailed in **Table 2**. This protocol utilized compression equipment (Yi Dong Kang Intelligent Compression Training Device, China), with 5 cm-wide compression bands applied at the upper third of the thigh to restrict blood flow velocity. FT was performed under these conditions. The CG followed a training protocol as outlined in **Table 3**, with all athletes in this group maintaining identical training content, sets, repetitions, and rest intervals to the EG. The only differences were the absence of BFR and variations in external load intensity. Both groups trained three times per week for a total of 24 weeks. In addition to these three weekly intervention training sessions, athletes in both groups uniformly participated in 5 to 6 weekly sessions of specialized technical and tactical training, with identical content across both groups.

**Table 2:** Weekly intervention plan for the EG.

Weekly training schedule	Exercise protocol	Sets and repetitions	Training load intensity	Rest interval	Bundled pressure	Inflation pressure
First Session	Single-Leg Romanian Deadlift	3 sets of 15 reps	30%1RM	1 min	40 mmHg	200 mmHg
	Bulgarian Single-Leg Squat	3 sets of 15 reps	30%1RM	1 min	40 mmHg	200 mmHg
	Bounce Squat	3 sets of 15 reps	30%1RM	1 min	40 mmHg	200 mmHg
	Weighted Plank on BOSU Ball with High Kick	3 sets of 15 reps	30%1RM	1 min	40 mmHg	200 mmHg
Second Session	Front-to-Back Split Squat	3 sets of 15 reps	30%1RM	1 min	40 mmHg	200 mmHg
	Squat with Forward Neck Flexion	3 sets of 15 reps	30%1RM	1 min	40 mmHg	200 mmHg
	Bulgarian Single-Leg Squat	3 sets of 15 reps	30%1RM	1 min	40 mmHg	200 mmHg
	Side-to-Side Split Squat on BOSU Ball with Barbell Plates	3 sets of 15 reps	30%1RM	1 min	40 mmHg	200 mmHg
Third Session	Half-Squat on Balance Pad	3 sets of 15 reps	30%1RM	1 min	40 mmHg	200 mmHg
	Bounce Squat	3 sets of 15 reps	30%1RM	1 min	40 mmHg	200 mmHg
	Single-Leg Romanian Deadlift	3 sets of 15 reps	30%1RM	1 min	40 mmHg	200 mmHg
	Weighted Plank with BOSU Ball and High Kick	3 sets of 15 reps	30%1RM	1 min	40 mmHg	200 mmHg

**Table 3:** Weekly intervention plan for the CG.

Weekly training schedule	Exercise protocol	Sets and repetitions	Training load intensity	Rest interval
First Session	Single-Leg Romanian Deadlift	3 sets of 15 reps	70%1RM	1 min
	Bulgarian Single-Leg Squat	3 sets of 15 reps	70%1RM	1 min
	Bounce Squat	3 sets of 15 reps	70%1RM	1 min
	Weighted Plank on BOSU Ball with High Kick	3 sets of 15 reps	70%1RM	1 min
Second Session	Front-to-Back Split Squat	3 sets of 15 reps	70%1RM	1 min
	Squat with Forward Neck Flexion	3 sets of 15 reps	70%1RM	1 min
	Bulgarian Single-Leg Squat	3 sets of 15 reps	70%1RM	1 min
	Side-to-Side Split Squat on BOSU Ball with Barbell Plates	3 sets of 15 reps	70%1RM	1 min
Third Session	Half-Squat on Balance Pad	3 sets of 15 reps	70%1RM	1 min
	Bounce Squat	3 sets of 15 reps	70%1RM	1 min
	Single-Leg Romanian Deadlift	3 sets of 15 reps	70%1RM	1 min
	Weighted Plank with BOSU Ball and High Kick	3 sets of 15 reps	70%1RM	1 min

To ensure the rigor of the experimental results and reduce the confounding effect of the “dynamic growth effect” on training outcomes, a one-repetition maximum (1RM) test was administered to all subjects every four weeks. Based on these test results, training load intensities (i.e., the weights lifted) were readjusted for the EG (30% 1RM) and CG (70% 1RM). Given that the movements were performed under non-stable conditions, direct measurement was employed instead of using a linear sensor system (GYMAWARE) to ensure precision. Initial 1RM values were estimated from recent training records or pretests involving multiple repetitions to failure. A progressive loading protocol was then followed: 50% of estimated 1RM for 8 repetitions, 70% for 5 repetitions, and 90% for 1 repetition, with rest intervals of 1, 2, and 3 minutes, respectively (Stratton et al., 2024). Formal testing began at 90% of the estimated 1RM, with loads progressively increased by 2%–10% (5%–10% for compound exercises, 2.5%–5% for single-joint exercises). A single repetition was performed at each load, followed by 2–4 minutes of rest to allow for full phosphagen system recovery (Haff and Triplett, 2015). No more than five attempts were allowed to avoid neuromuscular fatigue. The load of the last successful repetition, meeting the required standard, was recorded as the 1RM. Movement validity was assessed by a CSCS-certified specialist using the “all-or-nothing” principle; repetitions with insufficient range of motion, compensatory movement, or use of momentum were considered invalid.

A series of athletic performance and physical anthropometric measurements were conducted for all subjects at the beginning and end of the experiment, as well as at 4-week intervals (pretest, Week 4, Week 8, Week 12, Week 16, Week 20, Week 24), totaling seven measurement sessions.

### 2.5 Statistical analysis

Data were processed and analyzed using Microsoft Excel 2021 and SPSS 29.0 statistical software. All results are presented as mean ± standard deviation (*M* ± *SD*). Repeated measures analysis of variance (ANOVA) was used to examine changes in athletic performance and body composition at the seven time points before and after the intervention. The Shapiro-Wilk test was first applied to assess the normality of the data, and the Levene test was used to evaluate the homogeneity of variance. The main effects of time, the between-group main effect, and the interaction effects of time and group were tested sequentially using repeated measures ANOVA. The Greenhouse-Geisser correction was applied when the assumption of homogeneity of variances was violated. *Post-hoc* comparisons were conducted using the Bonferroni method. If the interaction effect was significant, simple effects analysis was performed, including (1) between-group comparisons at each time point and (2) within-group comparisons at each time point relative to baseline for both groups. If the interaction effect was not significant, pairwise comparisons for the main effects of time were conducted at all time points. Statistical significance was set at *P* < 0.05. Effect sizes were reported as partial *η*², with 0.01 representing a small effect, 0.06 a medium effect, and 0.14 a large effect.

## 3 Results

### 3.1 Changes in athletic performance

#### 3.1.1 Changes in maximum strength

Repeated measures analysis of variance (ANOVA) conducted on relevant experimental data after the conclusion of the experiment revealed a significant main effect of time (*F* = 537.820, *P <* 0.01, partial *η²* = 0.964), a significant main effect of group (*F* = 6.411, *P =* 0.02, partial *η²* = 0.243), and a significant interaction effect between time and group (*F* = 43.975, *P <* 0.01, partial *η²* = 0.687). Within−group comparisons (relative to baseline) revealed that 1RM half−squat performance in both the EG and CG was significantly higher than the pre−test level from week 4 onward (*P* < 0.01), and this elevation persisted through week 24. Between-group comparisons at the same time points revealed that a significant group effect emerged from week 8 through week 24, with the EG demonstrating a greater magnitude of improvement than the CG (week 8, *P* = 0.02; weeks 12–24, *P* < 0.01).

**Figure 3:**
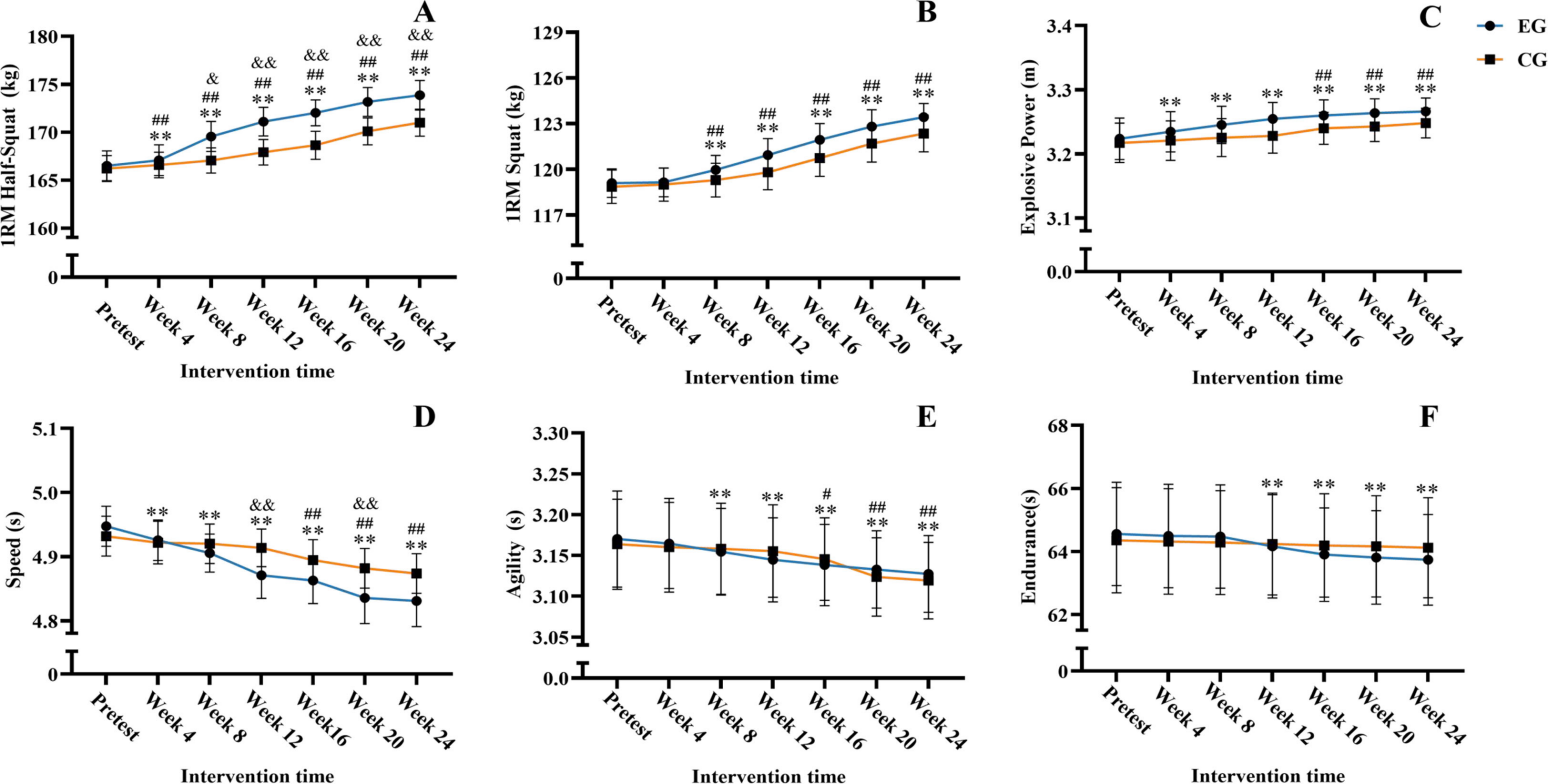
Trend chart of changes in physical performance. Six-panel line graph compares experimental group (EG, blue) and control group (CG, orange) over 24 weeks for 1RM half-squat **(A)**, 1RM squat **(B)**, explosive power **(C)**, speed **(D)**, agility **(E)**, and endurance **(F)**. EG generally shows greater performance improvements across all metrics compared to CG, with statistical significance markers indicating differences and changes over time. Within-group comparisons between baseline and each time point are indicated by **P < 0.01 for EG, and by ^#^*P* < 0.05 and ^##^*P* < 0.01 for CG. Between-group differences are indicated by ^&&^*P* < 0.01.

Analysis of the 1RM squat revealed a significant main effect of time (*F* = 351.945, *P <* 0.01, partial *η²* = 0.946), while the main effect of group was not significant (*F* = 1.440, *P =* 0.24, partial *η²* = 0.067). A significant interaction effect between time and group was observed (*F* = 7.409, *P <* 0.01, partial *η²* = 0.270). As presented in **Table 4** and **Figures 3A, B**, significant improvements relative to baseline were observed in both the EG and CG by week 8 of training (*P* < 0.01). Intergroup comparisons at identical time points showed no statistical significance (*P >* 0.05).

**Table 4:** Changes in various athletic performance after training intervention.

Project	Group	Pretest	Week 4	Week 8	Week 12	Week 16	Week 20	Week 24	Interaction effect F-value	Main effect	
										Group	Time
1RM Half- Squat (kg)	EG	166.5 ± 2.3	167.1 ± 2.4**	169.6 ± 2.3**^&^	171.1 ± 2.2**^&&^	172.0 ± 2.0**^&&^	173.2 ± 2.2**^&&^	173.9 ± 2.3**^&&^	42. 771^△^	6.277^△^	436.393^△^
	CG	166.2 ± 2.0	166.6 ± 2.0^##^	167.1 ± 2.0^##^	167.9 ± 2.0^##^	168.6 ± 2.2^##^	170.1 ± 2.1^##^	171.0 ± 2.1^##^			
1RM Squat (kg)	EG	119.1 ± 1.4	119.1 ± 1.4	120.0 ± 1.4**	120.9 ± 1.6**	121.9 ± 1.6**	122.8 ± 1.6**	123.4 ± 1.3**	7.409^△^	1.440	351.945^△^
	CG	118.9 ± 1.6	119.0 ± 1.6	119.3 ± 1.7^##^	119.8 ± 1.7^##^	120.7 ± 1.8^##^	121.7 ± 1.8^##^	122.4 ± 1.8^##^			
Explosive Power (m)	EG	3.22 ± 0.04	3.23 ± 0.05**	3.25 ± 0.04**	3.25 ± 0.04**	3.26 ± 0.04**	3.26 ± 0.03**	3.27 ± 0.03**	2.555	1.143	47.655^△^
	CG	3.22 ± 0.05	3.22 ± 0.05	3.23 ± 0.04	3.23 ± 0.04	3.24 ± 0.04^##^	3.24 ± 0.03^##^	3.23 ± 0.06^##^			
Speed (s)	EG	4.95 ± 0.05	4.93 ± 0.05**	4.91 ± 0.04**	4.87 ± 0.05**^&&^	4.86 ± 0.05**	4.84 ± 0.06**^&&^	4.83 ± 0.06**	15.240^△^	1.258	108.796^△^
	CG	4.93 ± 0.05	4.92 ± 0.05	4.92 ± 0.05	4.91 ± 0.04	4.89 ± 0.05^##^	4.88 ± 0.05^##^	4.87 ± 0.05^##^			
Agility (s)	EG	3.17 ± 0.09	3.16 ± 0.08	3.15 ± 0.08**	3.14 ± 0.08**	3.14 ± 0.07**	3.13 ± 0.07**	3.13 ± 0.07**	2.608	0.001	45.167^△^
	CG	3.16 ± 0.08	3.16 ± 0.08	3.16 ± 0.08	3.16 ± 0.08	3.15 ± 0.08^#^	3.12 ± 0.07^##^	3.12 ± 0.07^##^			
Endurance (s)	EG	64.55 ± 2.44	64.49 ± 2.44	64.47 ± 2.44	64.16 ± 2.44**	63.90 ± 2.21**	63.81 ± 2.21**	63.74 ± 2.14**	7.833	0.006	20.709^△^
	CG	64.35 ± 2.48	64.32 ± 2.49	64.28 ± 2.45	64.24 ± 2.41	64.19 ± 2.44	64.16 ± 2.39	64.12 ± 2.36			

Comparisons between baseline values and measurements at each intervention time point within the EG are indicated by * (*P* < 0.05) and ** (*P* < 0.01), while corresponding comparisons within the CG are denoted by ^#^(*P* < 0.05) and ^##^ (*P* < 0.01). Between-group differences between the EG and CG at the same intervention time point are represented by ^&^ (*P* < 0.05) and ^&&^ (*P* < 0.01). A significant Main Effect of group, time, or their Interaction Effect is indicated by ^Δ^ (*P* < 0.05).

#### 3.1.2 Changes in explosive power

Analysis of explosive power (one-step approach vertical jump) revealed a significant main effect of time (*F* = 47.655, *P <* 0.01, partial *η²* = 0.704), while the main effect of group (*F* = 1.143, *P =* 0.30, partial *η²* = 0.054) and the interaction effect between time and group (*F* = 2.555, *P =* 0.09, partial *η²* = 0.113) were both insignificant. Since the interaction effect was insignificant, *post hoc* tests were conducted on the significant main effect of time. As presented in **Table 4** and **Figure 3C**, the vertical reach height of all subjects was significantly higher than the pretest measurement starting from week 4 (*P <* 0.01) and remained so through week 24. Significant improvements were observed in the CG from week 16 of training onward (*P* < 0.01). No significant differences in vertical reach height were observed between groups at different intervention time points (*P >* 0.05).

#### 3.1.3 Speed changes

Analysis of speed (30m sprint) after the intervention revealed a significant main effect of time (*F* = 108.796, *P <* 0.01, partial *η²* = 0.845), while the main effect of group was not significant (*F* = 1.258, *P =* 0.28, partial *η²* = 0.059). The interaction effect between group and time was significant (*F* = 15.240, *P <* 0.01, partial *η²* = 0.432). Intragroup comparisons revealed that the EG’s 30m sprint performance significantly surpassed their pretest scores starting from Week 4 (*P <* 0.01). The CG’s performance showed significant improvement only from Week 16 onwards (*P <* 0.01). Between−group comparisons revealed that the 30−m sprint performance of the EG was significantly superior to that of the CG at weeks 12 and 20 (*P* < 0.01). Detailed changes in speed performance are presented in **Table 4** and **Figure 3D**.

#### 3.1.4 Agility changes

Analysis of specialized agility changes (three-second zone agility) revealed a significant main effect of time (*F* = 45.167, *P* < 0.01, partial *η²* = 0.693). The main effect of group was not significant (*F* = 0.001, *P =* 0.978, partial *η²* = 0.000), and the interaction effect between group and time was not significant (*F* = 2.608, *P =* 0.103, partial *η²* = 0.115). As presented in **Table 4** and **Figure 3E**, relative to baseline, the experimental group (EG) exhibited statistically significant improvements from weeks 8 to 24 (*P* < 0.01). In the CG, significant improvements were observed at week 16 (*P* = 0.04) and from weeks 20 to 24 (*P* < 0.01). No statistically significant differences were found between groups at any time point (*P >* 0.05).

#### 3.1.5 Endurance changes

Analysis of specialized endurance changes (17-return shuttle run) revealed a significant main effect of time (*F* = 20.709, *P <* 0.01, partial *η²* = 0.509), a non-significant main effect of group (*F* = 0.006, *P =* 0.94, partial *η²* = 0.000), and the interaction effect between group and time was significant (*F* = 7.833, *P <* 0.01, partial *η²* = 0.281). As presented in **Table 4** and **Figure 3F**, endurance performance in the EG showed significant improvements from week 12 onward relative to baseline (*P* < 0.01), with this enhancement persisting through week 24. No significant changes were observed in the CG (*P* > 0.05). No statistically significant differences were found between groups at any time point (*P >* 0.05).

### 3.2 Changes in body composition

#### 3.2.1 Weight changes

**Figure 4:**
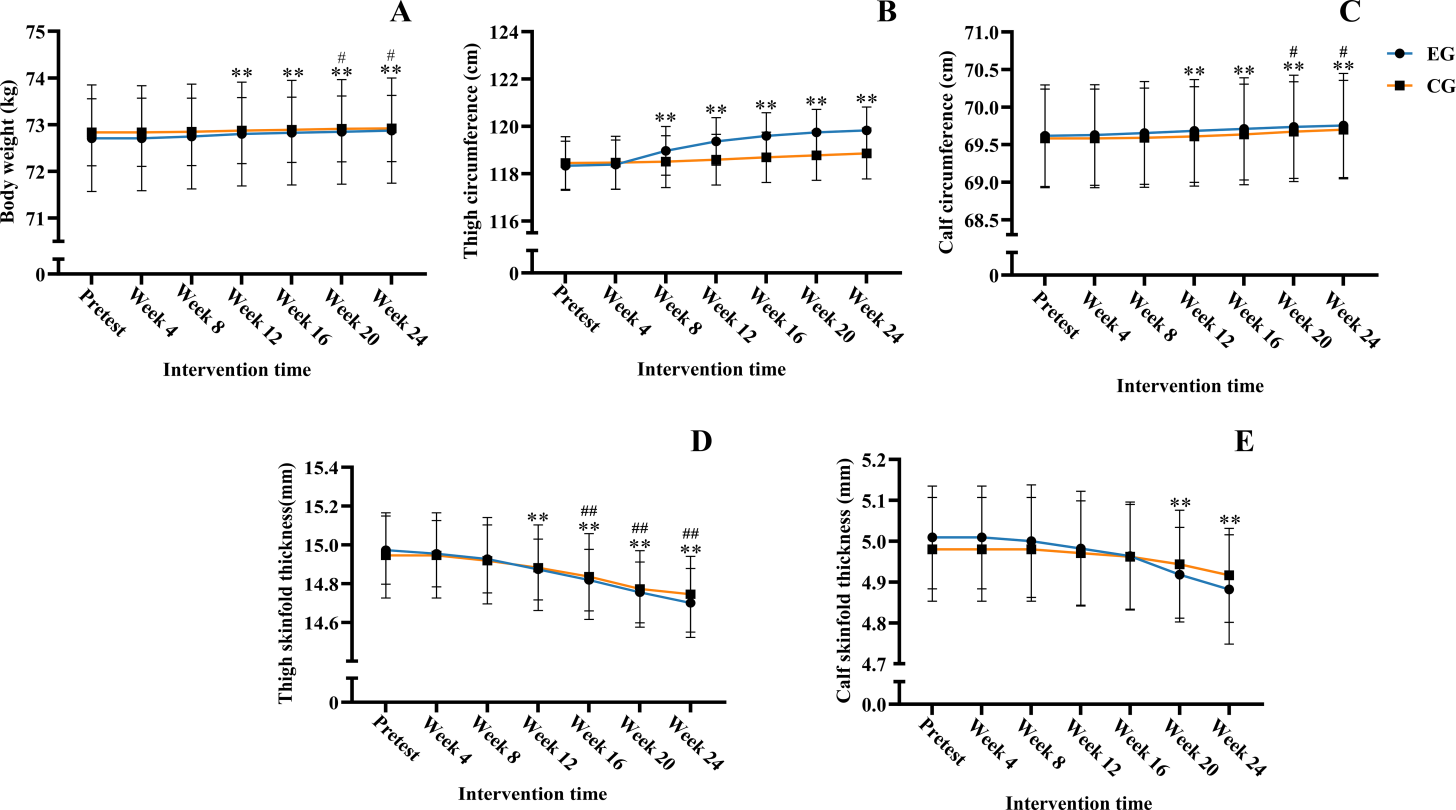
Body shape trend chart. Five line graphs labeled G through K compare body weight, thigh circumference, calf circumference, thigh skinfold thickness, and calf skinfold thickness between EG and CG groups across multiple intervention times. Significant differences are indicated by asterisks and number signs with error bars shown for each data point.Within-group comparisons between baseline and each time point are indicated by **P < 0.01 for EG, and by ^#^*P* < 0.05 and ^##^*P* < 0.01 for CG.

Repeated measures ANOVA revealed a significant main effect of time (*F* = 25.966, *P <* 0.01, partial *η²* = 0.565), while the main effect of group was not significant (*F* = 0.021, *P =* 0.89, partial *η²* = 0.001). The interaction effect between group and time was significant (*F* = 2.750, *P =* 0.04, partial *η²* = 0.121). Intragroup comparisons revealed that the EG’s weight was significantly higher than the pre-experiment measurement starting from week 12 (*P <* 0.01); the CG’s weight did not show a significant increase until week 20 (*P =* 0.03) and week 24 (*P =* 0.02). Intergroup comparisons showed that, despite differing pacing of changes, no intergroup comparisons at any identical time point reached statistical significance (*P >* 0.05). Weight change trends is shown in **Table 5** and **Figure 4A**.

**Table 5:** Changes in body morphology after training intervention.

Project	Group	Pretest	Week 4	Week 8	Week 12	Week 16	Week 20	Week 24	Interaction effect F-value	Main effect	
										Group	Time
Body weight (kg)	EG	72.7 ± 1.7	72.7 ± 1.7	72.8 ± 1.7	72.8 ± 1.7**	72.8 ± 1.7**	72.9 ± 1.7**	72.9 ± 1.7**	2.750^△^	0.021	25.966^△^
	CG	72.8 ± 1.1	72.8 ± 1.1	72.9 ± 1.1	72.9 ± 1.1	72.9 ± 1.0	72.9 ± 1.1^#^	72.9 ± 1.1^#^			
Thigh circumference(cm)	EG	118.3 ± 1.6	118.4 ± 1.6	119.0 ± 1.5**	119.4 ± 1.5**	119.6 ± 1.5**	119.8 ± 1.4**	119.8 ± 1.5**	21.072^△^	0.713	54.468^△^
	CG	118.5 ± 1.7	118.5 ± 1.7	118.5 ± 1.6	118.6 ± 1.6	118.7 ± 1.6	118.8 ± 1.6	118.9 ± 1.6			
Calf circumference (cm)	EG	69.6 ± 1.0	69.6 ± 1.0	69.7 ± 1.0	69.7 ± 1.0**	69.7 ± 1.0**	69.7 ± 1.0**	69.8 ± 1.0**	0.860	0.019	44.588^△^
	CG	69.6 ± 1.0	69.6 ± 1.0	69.6 ± 1.0	69.6 ± 1.0	69.6 ± 1.0	69.7 ± 1.0^#^	69.7 ± 1.0^#^			
Thigh skin fold thickness (mm)	EG	15.0 ± 0.3	15.0 ± 0.3	14.9 ± 0.3	14.9 ± 0.2**	14.8 ± 0.2**	14.8 ± 0.2**	14.7 ± 0.3**	1.117	0.003	67.018^△^
	CG	15.0 ± 0.3	15.0 ± 0.3	14.9 ± 0.3	14.9 ± 0.3	14.8 ± 0.3^##^	14.8 ± 0.3^##^	14.8 ± 0.3^##^			
Thickness of calf skin folds (mm)	EG	5.0 ± 0.2	5.0 ± 0.2	5.0 ± 0.2	5.0 ± 0.2	5.0 ± 0.2	4.9 ± 0.2**	4.9 ± 0.2**	3.524^△^	0.003	28.790^△^
	CG	5.0 ± 0.2	5.0 ± 0.2	5.0 ± 0.2	5.0 ± 0.2	4.9 ± 0.2	4.9 ± 0.2	4.9 ± 0.2			

Comparisons between baseline values and measurements at each intervention time point within the EG are indicated by ^*^(*P* < 0.05) and ^**^(*P* < 0.01), while corresponding comparisons within the CG are denoted by ^#^(*P* < 0.05) and ^##^(*P* < 0.01). Between-group differences between the EG and CG at the same intervention time point are represented by ^&^(*P* < 0.05) and ^&&^(*P* < 0.01). A significant Main Effect of group, time, or their Interaction Effect is indicated by ^△^(*P* < 0.05).

#### 3.2.2 Changes in lower limb circumference

Analysis of thigh circumference revealed a significant main effect of time (*F* = 54.468, *P <* 0.01, partial *η²* = 0.731), while the main effect of group was insignificant (*F* = 0.713, *P =* 0.41, partial *η²* = 0.034). while the interaction effect between group and time was significant (*F* = 24.072, *P <* 0.01, partial *η²* = 0.513). Intragroup comparisons revealed that the thigh circumference of the EG was significantly higher than the pretest measurement starting from week 8 (*P <* 0.01) and continued to increase. In contrast, the thigh circumference of the CG showed no significant change from the pretest throughout the intervention period (*P* > 0.05). Between-group comparisons revealed that although the pace of change differed, no statistically significant differences were found at any identical time point (*P >* 0.05).

Analysis of calf circumference showed a significant main effect of time (*F* = 44.588, *P <* 0.01, partial *η²* = 0.690), while the group main effect was not significant (*F* = 0.019, *P =* 0.89, partial *η²* = 0.001). The interaction between group and time was also not significant (*F* = 0.860, *P =* 0.49, partial *η²* = 0.041). *Post hoc* analysis of the significant main effect of time revealed that calf circumference measurements in the EG were significantly higher than baseline values from week 12 onward (*P* < 0.01), whereas no significant changes were observed in the CG (*P* > 0.05). No statistically significant differences were observed between groups at any time point (*P >* 0.05). Trends in thigh and calf circumference changes are shown in **Table 5**, **Figures 4B, C**.

#### 3.2.3 Changes in lower limb skin fold thickness

Repeated measures ANOVA of thigh skin fold thickness revealed a significant main effect of time (*F* = 67.018, *P* < 0.01, partial *η²* = 0.770), but neither the main effect of group (*F* = 0.003, *P* = 0.96, partial *η²* = 0.000) nor the interaction effect between time and group (*F* = 1.117, *P* = 0.35, partial *η²* = 0.053) was not significant. *Post hoc* analysis of the significant main effect of time revealed that thigh skinfold thickness in the EG was significantly lower than baseline values from week 12 onward (*P* < 0.01), and this reduction persisted through week 24. In the CG, thigh skinfold thickness was significantly lower than baseline values from week 16 onward (*P* < 0.01). No statistically significant between-group differences were observed at any time point (*P* > 0.05).

Analysis of calf skinfold thickness revealed a significant main effect of time (*F* = 28.790, *P <* 0.01, partial *η²* = 0.590), but no significant main effect of group (*F* = 0.003, *P =* 0.96, partial *η²* = 0.000). while the interaction effect between time and group was significant (*F* = 3.524, *P =* 0.02, partial *η²* = 0.150). Intragroup comparisons revealed that calf skinfold thickness in the EG was significantly lower than the pre-experiment measurement starting from week 20 (*P <* 0.01), whereas changes in the CG were not significant (*P >* 0.05). Between-group comparisons revealed that although the patterns of change differed, no statistically significant differences were found between groups at any identical time point (*P >* 0.05).

The trends in changes of thigh and calf skinfold thickness are shown in **Table 5**, **Figures 4D, E**.

## 4 Discussion

### 4.1 Changes in athletic performance

#### 4.1.1 Changes in strength performance

As shown in **Table 4**, after 24 weeks of FT and BFR−FT, subjects showed significant improvements in 1RM half-squat (weeks 4–24) and 1RM squat (weeks 8–24) compared to baseline (*P* < 0.01). Regarding explosive power, both the EG (weeks 4–24) and the CG (weeks 16–24) showed significant increases in the one-step approach vertical jump relative to pre-intervention values (*P* < 0.01). Numerous studies have indicated that FT effectively enhances lower-body muscle strength in athletes, including volleyball players (Gao et al., 2025), weightlifters (Adami et al., 2022), hockey players (Gürkan et al., 2025), and soccer players (Niknam et al., 2025). Similarly, BFR has been shown to enhance lower-body strength in soccer players (Korkmaz et al., 2022), rugby players (Takarada et al., 2002), and wrestlers (Jiang et al., 2025). Da Silva-Grigoletto et al. (2020) demonstrated that incorporating BFR into FT further augments training outcomes. Lyu et al. (2024) observed significant improvements in lower-body knee peak torque and explosive power (vertical jump, squat jump) when applying BFR-FT to male soccer players, with gains exceeding those achieved through functional strength training alone. Likewise, a study on elite female soccer players showed that this training method effectively enhanced isometric strength and explosive power (standing long jump, running vertical jump), while also reducing lower-limb strength asymmetry and excessive bilateral strength imbalances (Lyu et al., 2024). The results of the current study align closely with these findings.

Furthermore, the most rapid increases in 1RM half-squat were observed during weeks 4–8 in the EG and weeks 16–20 in the CG. For 1RM squat, the greatest gains occurred between weeks 4–20 (EG) and weeks 12–20 (CG). The most pronounced increases in explosive power occurred during the first 12 weeks in the EG and between weeks 12–16 in the CG. These results suggest that BFR-FT produces both a “faster onset” and “greater magnitude” of strength gains compared to FT alone. This could be attributed to the effects on oxygen content; BFR slows blood velocity, reducing mitochondrial reactive oxygen species (ROS) in skeletal muscle while increasing related metabolic byproducts. This shift prioritizes the recruitment of fast-twitch (Type II) fibers over slow-twitch (Type I) fibers, which promotes greater muscle strength development (Che, 2021; Loenneke et al., 2010; Petrick et al., 2019). BFR-FT specifically induces fast-twitch fiber recruitment, thereby providing stronger targeted effects and greater stimulation. This may be a key reason why BFR-FT enhances maximal strength and explosive power more rapidly than FT alone.

Strength gains also involve neural adaptations and increases in muscle cross-sectional area (Deng et al., 2015). FT enhances strength performance by stimulating deep muscles through multidimensional training under unstable conditions, thereby improving neuromuscular coordination (Yin and Yuan, 2015). However, some studies suggest that for highly trained athletes or those with long-term training experience, the neural pathway to strength gains has less impact compared to increases in muscle cross-sectional area (Ratamess, 2012). While no significant difference was observed between low-load BFR-FT and high-load FT in the current study, this finding suggests that the low-load approach can produce effects comparable to those achieved with high-load training. Analysis of body composition changes revealed increased body weight, greater thigh circumference, and reduced skinfold thickness, suggesting that the 24-week training regimen likely increased thigh muscle mass. Consequently, the greater improvements in maximal strength and power observed in the EG appear to support Ratamess’s conclusion regarding the importance of muscle mass in strength development. These findings are particularly significant for enhancing strength performance in basketball players.

#### 4.1.2 Changes in speed, agility, and endurance performance

As shown in **Table 4** and **Figure 2**, the EG exhibited a significant improvement in speed from week 4 to week 24 (*P* < 0.01), while the CG showed a marked improvement from week 16 to week 24 (*P* < 0.01). Agility in the EG significantly improved from week 8 to week 24 (*P* < 0.01), whereas the CG showed significant enhancements from week 16 to week 24 (week 16, *P* = 0.04; weeks 20–24, *P* < 0.01). Endurance in the EG demonstrated significant variation from week 12 to week 24 (*P* < 0.01), while no significant changes were observed in the CG (*P* > 0.05). Related studies indicate that FT effectively enhances speed (Baron et al., 2019; Bieniec and Grabara, 2025; Keiner et al., 2022) and agility (Khazaei et al., 2023; Bieniec and Grabara, 2025; Gao et al., 2025). BFR has also been shown to improve speed performance (Baron et al., 2019; Castilla-López and Romero-Franco, 2023). Furthermore, BFR-FT studies have demonstrated significant improvements in 30-m sprint and T-agility test scores among both male and female soccer players (Lyu et al., 2023, 2024). The findings of this study are consistent with previous research and highlight the significance of these results for enhancing athletic performance in basketball players.

Moreover, the EG demonstrated significantly greater improvements in speed compared to the CG. At each intervention time point throughout the 24-week period, the EG consistently outperformed the CG. While no significant between-group differences in agility were observed, the results indicate that the training effects induced by low-load BFR-FT were comparable to those achieved with high-load FT. Analysis of the two training methods suggests that this difference may stem from the unique physiological load stimulus of BFR, which specifically induces and stimulates fast-twitch muscle fibers. These fibers generate greater force during contraction (Che, 2021; Deng et al., 2015; De Castro et al., 2024), thereby exerting a greater influence on speed and agility performance. Existing research rarely reports FT inducing specific effects on a particular muscle fiber type, which may explain the more pronounced differences in gains between the two training methods.

Additionally, the EG showed a significant improvement in endurance, which may be attributed to the physiological hypoxia induced by BFR. Research on hypoxic training has shown that hypoxia accelerates oxygen delivery and utilization following ischemia, enhances regulation of local blood flow and oxygen-dependent metabolic pathways, and improves oxygen redistribution and utilization efficiency—all contributing to enhanced endurance performance (Kos et al., 2009). Findings from studies on skeletal muscle oxygenation during hypoxic training support this notion, demonstrating that sustained hypoxia significantly enhances skeletal muscle oxygenation and hemodynamic parameters, confirming hypoxia’s role in improving oxygen utilization efficiency and microvascular function (Zhou et al., 2026). The 17-return shuttle run endurance test used in this study is specific to basketball. Given its testing requirements and duration, it qualifies as a mixed anaerobic-aerobic endurance test. The EG’s training method may have been more conducive to enhancing anaerobic endurance, which could explain the observed performance gains. Lyu et al. (2023) found that BFR-FT significantly increased athletes’ maximal anaerobic power and average anaerobic power. However, whether BFR-FT affects aerobic endurance, and which type of endurance (aerobic or anaerobic) it influences more significantly, remains an area for further investigation.

### 4.2 Changes in body composition

The results related to body composition changes following BFR-FT indicated increases in thigh muscle mass between weeks 12 and 24, and in calf muscle mass between weeks 20 and 24 (*P* < 0.05). In contrast, FT alone did not result in significant increases in either thigh or calf muscle mass (*P* > 0.05). These changes were inferred from three indicators: body weight, limb circumference, and skinfold thickness. Given the absence of significant interaction effects, the above findings are based solely on descriptive data and should not be interpreted as statistically conclusive. Gürkan et al. (2025) reported that 12 weeks of FT improved strength performance and body composition (increased weight and BMI, decreased body fat) in elite male hockey players, but did not specify which muscle groups gained mass. Sánchez-Valdepeñas et al. (2025) demonstrated that squats with BFR effectively enhanced muscle hypertrophy. Other studies also show that BFR significantly increases lower-limb muscle mass (De Lemos Muller et al., 2024). The present findings are consistent with these observations, demonstrating that BFR-FT significantly promotes increases in thigh muscle mass in basketball players. This effect may be tied to load intensity, a key determinant of strength development in resistance training. Gains from BFR at 20–40% 1RM combined with resistance training are comparable to those achieved with 70% 1RM (Che, 2021). Leading organizations such as the ACSM and NSCA recommend resistance training at no less than 70% 1RM for optimal hypertrophy and strength gains. Lower intensities (< 70% 1RM) provide less stimulus and result in reduced muscle protein synthesis (American College of Sports Medicine, 2009).

Moreover, BFR enhances muscular blood flow and promotes the accumulation of metabolic byproducts, such as lactate, within muscle cells. This, in turn, stimulates the secretion of anabolic hormones, including growth hormone and myostatin, and promotes the synthesis of myosin (Palmieri-Smith et al., 2022; Takarada et al., 2000b; Takarada et al., 2002). BFR also stimulates protein synthesis by activating the Akt/mTOR signaling pathway, thereby increasing muscle mass (Loenneke et al., 2010). Pazokian et al. (2022) observed that BFR enhanced follistatin levels and decreased myostatin levels, further amplifying these biomarker expressions when integrated with FT. This finding supports the conclusion that BFR-FT yields superior muscle mass gains compared to FT alone. Moreover, restricting blood flow specifically stimulates fast-twitch muscle fibers, which are more responsive to resistance training than slow-twitch fibers, thereby increasing muscle cross-sectional area (Che, 2021; Deng et al., 2015; Takarada et al., 2000a; Takarada et al., 2002). However, this study did not observe FT increasing lower-body muscle mass in athletes. This discrepancy may stem from the nature of FT, which primarily enhances neuromuscular coordination and control, rather than muscle cross-sectional area. It may also relate to the athletic level and prior training experience of the subjects, both of which require further investigation.

## 5 Limitations

Although the present study has confirmed the positive effects of a 24-week BFR−FT intervention on athletic performance and body composition in high-level basketball players, several limitations warrant consideration. First, the sample size was relatively small due to the challenges associated with recruiting high-level athletes, despite meeting the minimum requirement based on power analysis. Second, while overall statistical power was confirmed *post hoc*, the current sample size may still be underpowered to detect group-by-time interaction effects, particularly given the multiple repeated measurements and the inherent complexity of detecting such interactions. Third, due to experimental constraints, only indirect methods were used to infer body composition, which limited the ability to accurately assess muscle mass and associated physiological mechanisms. Therefore, future research should aim to incorporate larger sample sizes and more precise measurement instruments to further substantiate the findings. Additionally, the applicability of this training approach to athletes from other sports or varying competitive levels remains to be explored and warrants further investigation.

## 6 Conclusions

The 24-week BFR-FT intervention effectively enhances lower-limb athletic performance metrics, including maximal strength, explosive power, speed, agility, and endurance. These improvements are accompanied by favorable changes in body composition, such as increases in body weight and thigh circumference, as well as reductions in thigh skinfold thickness. FT alone significantly improves maximal strength, explosive power, speed, and agility, but has limited effects on endurance performance and body composition variables. BFR-FT, however, exhibits a notable group-by-time interaction effect on 1RM half-squat and speed performance, with superior improvements observed in comparison to the CG. For all other measured variables, both groups demonstrated a significant main effect of time, but no between-group differences were observed. The combined BFR-FT approach offers a viable alternative or adjunct to high-load FT protocols. The findings from this 24-week study provide valuable insights into the efficacy and specific effects of FT, with or without BFR, in optimizing athletic performance and body composition in high-level basketball players. These results offer practical guidance for coaches and serve as empirical evidence to inform future research in this domain.

## Data Availability

The original contributions presented in the study are included in the article/Supplementary Material. Further inquiries can be directed to the corresponding authors.
